# Insulin Resistance as a Shared Pathogenic Mechanism Between Depression and Type 2 Diabetes

**DOI:** 10.3389/fpsyt.2019.00057

**Published:** 2019-02-14

**Authors:** Natalia de M. Lyra e Silva, Minh P. Lam, Claudio N. Soares, Douglas P. Munoz, Roumen Milev, Fernanda G. De Felice

**Affiliations:** ^1^Centre for Neuroscience Studies, Queen's University, Kingston, ON, Canada; ^2^Department of Psychiatry, Queen's University, Kingston, ON, Canada; ^3^Institute of Medical Biochemistry Leopoldo De Meis, Federal University of Rio de Janeiro, Rio de Janeiro, Brazil

**Keywords:** depression, type 2 diabetes, insulin resistance, inflammation, synaptic plasticity, hippocampus, HPA axis, dopamine

## Abstract

Neuropsychiatric disorders and type 2 diabetes (T2D) are major public health concerns proposed to be intimately connected. T2D is associated with increased risk of dementia, neuropsychiatric and mood disorders. Evidences of the involvement of insulin signaling on brain mechanisms related to depression indicate that insulin resistance, a hallmark of type 2 diabetes, could develop in the brains of depressive patients. In this article, we briefly review possible molecular mechanisms associating defective brain insulin signaling with reward system, neurogenesis, synaptic plasticity and hypothalamic-pituitary-adrenal (HPA) stress axis in depression. We further discuss the involvement of tumor necrosis factor α (TNFα) promoting defective insulin signaling and depressive-like behavior in rodent models. Finally, due to the high resistant rate of anti-depressants, novel insights into the link between insulin resistance and depression may advance the development of alternative treatments for this disease.

## Introduction

Depressive disorders, type 2 diabetes (T2D) and obesity are among the top causes of years lived with disability, a widely accepted measure of disease burden on society (1). Major Depressive Disorder represents the highest burden among mental disorders, with a significant impact on individuals, their families and the community at large. The World Health Organization estimates that more than 300 million people suffer from depressive disorders (2). In parallel, T2D has been estimated to afflict >400 million adults worldwide (2). T2D is characterized by peripheral insulin resistance that culminates in hyperglycemia (3). While T2D has precise diagnostic parameters, that includes fasting serum glucose levels and glycated hemoglobin blood levels; depression diagnoses is based on the persistence of some of the following symptoms for >2 weeks: sad/anxious mood, hopelessness, helplessness, decreased energy, appetite/weight changes, headaches, sleep changes, feelings of guilt, loss of interest, decreased concentration, psychomotor retardation and suicide attempts (4).

T2D is a risk factor not only for the development of cardiovascular diseases, but also to neurological and psychiatric disorders, including Alzheimer's disease (AD) (5, 6). Some clinical reports and meta-analyses indicate a correlation between T2D and depression with a bi-directional increased risk between both conditions (6–9). Albeit evidences correlating T2D and depression, confounders implicated in epidemiological studies hamper the temporal order assessment of those co-morbid cases. As a result, the association between both diseases is still unclear (8, 10, 11). Insulin signaling play a role in neuronal dysfunction and cognitive decline in AD (12, 13) and it emerged as a possible mechanism underlying alterations in the brain and in behavior in mood disorders (14). In children, the severity of depressive symptoms predicted the development of insulin resistance (15) and a recent report demonstrated that worst insulin resistance correlated with more pronounced depressive symptoms and dysfunction of the anterior cingulate cortex-hippocampal motivational network in a cohort of obese depressed youths (16).

In this minireview, we highlight a possible role of decreased insulin signaling in the brain, as a result of metabolic dyshomeostasis, in mechanisms involved with depressive behavior.

## Molecular Links Between Depression and Diabetes

### Brain Insulin Resistance

Insulin has been implicated with diverse central roles, like modulating feeding behavior and energy maintenance by the hypothalamus, as well as memory-related processes by the hippocampus (13, 17–19). Insulin receptors are expressed throughout the brain, including regions classically involved with mood regulation, such as the nucleus accumbens (NAc), the ventral tegmental area (VTA), the amygdala, and the raphe nuclei (20, 21). The knockdown of insulin receptors in the hypothalamus of rats triggered depressive and anxiety-like behaviors in mice (22). Anxiety and depressive-like behaviors were further reported in mice with neuronal-specific knockout of insulin receptors (NIRKO). NIRKO mice also exhibited mitochondrial dysfunction, oxidative stress and increased monoamine oxidase expression and dopamine turnover in the mesolimbic system (23). Interestingly, altered behavior was detected in 17-month-old NIRKO mice, but not in younger animals (23). It is important to note that by this age, these animals display increased white adipose tissue and plasma leptin concentration (17), raising the possibility of the behavior response being a secondary effect to the absence of insulin signaling in neurons.

A recent study demonstrated that the knockdown of insulin receptors in astrocytes also generates anxiety and depressive-like behavior in mice, via decreased purinergic signaling and altered dopamine release (24). A recent *post-mortem* analysis in the brain of patients diagnosed with mental illness has observed a correlation between gene expression of proteins involved with both the dopaminergic system and the insulin signaling (25), supporting the idea that insulin could regulate the dopaminergic response. Oppositely, another report observed that deleting insulin receptors from dopaminergic neurons had no impact on anxiety or depressive-like behavior in young adult mice (26). The absence of altered behavior in this model counteracts the idea of insulin regulating the dopaminergic system. Other possibilities to explain this phenotype are the development of compensatory mechanisms, or that, similar to what is observed in the NIRKO mice, altered behavior would be detected in older animals.

Defective brain insulin signaling in T2D patients has been associated with impaired transport of the hormone across the blood-brain barrier (27). Markers of impaired insulin signaling are present in the brain of *db/db* mice, a transgenic model for T2D that lacks the long isoform of the leptin receptor (28). These mice also exhibit increased immobility time in the forced swim test as early as 5 weeks of age, coinciding with an initial metabolic dysregulation, including hyperglycemia, increased food and water intake and body weight (29–31). This animal model also presents with progressive anxious and psychosis-like behavior that progress with age (30). Interestingly, since most metabolic parameters are also aggravated with aging in the *db/db* mice, it hinders an accurate determination of the major player influencing the behavior. High-fat diet (HFD) promotes T2D symptoms, as well as anxiety and depressive-like behavior in wild-type mice associated with impaired brain insulin signaling (32). Parallelly, HFD disrupts brain reward system of mice, by altering dopamine-related proteins in the VTA, NAc and dorsolateral striatum (32). Overall, further studies designed to investigate a direct correlation between brain insulin dysfunction and depressive-like behavior are needed in the field.

### Neurogenesis and Synaptic Plasticity

Hippocampal neurogenesis, a process in which neural progenitors from the subgranular zone differentiate into new neurons at the dentate gyrus, is proposed to be involved with depression and to be impaired in diabetes (33, 34). HFD impairs cell proliferation, insulin signaling and the Akt/glycogen synthase kinase 3β (GSK3β) activation promoted by serotonin in the dentate gyrus of the hippocampus. Interestingly, replacing HFD by chow diet recovered depressive symptoms and Akt/GSK3β response to insulin, even without a complete recovery of body weight. Neurogenesis was partially recovered by a chow diet replacement, suggesting that it was not the only mechanism implicated with the beneficial effect promoted by the regular diet (35). Other hormones like Insulin-like growth factor I (IGF-I) and leptin activate Akt and GSK3β pathway and mediate hippocampal neurogenesis (36–39). Interestingly, downregulation of those hormones are observed in the hippocampus of rodent models of T2D, being other possible targets to the link between T2D and depression (40, 41). Neurogenesis is also proposed to be impaired in T2D due to mitochondrial dysfunction (42). Peroxisome proliferator-activated receptor gamma (PPARγ) agonists increase central insulin sensitivity, mitochondrial biogenesis and prevent depressive-like behavior in rats through facilitation of hippocampal neurogenesis (43, 44).

Defective synaptic plasticity may lead to impairment of stress adaptation, prompting the onset of depression (45). In the food reward circuitry, insulin actions modulate synaptic plasticity in a concentration, time and brain region -dependent manner [for a review see (46)]. For instance, insulin promotes long-term depression of glutamatergic afferent connections into the VTA (47), but increases the activity of striatal cholinergic interneurons, elevating dopamine release into the NAc (48). Downregulation of insulin receptors in the hippocampus of rats impaired proper long-term potentiation response mediated by high frequency stimulation and decreased glutamate receptors levels (19). This approach also worsened learning behavior in a similar fashion to what is observed in T2D rodent models (19). Altogether, data indicate that brain insulin resistance can impair physiological mechanisms of reward and learning that would ultimately elicit depressive symptoms.

### Hypothalamic-Pituitary-Adrenal (HPA) Axis

Chronic psychological stress is associated with neuropsychiatric diseases, including depression and also with T2D (49–51). A well-supported theory of depression and T2D pathophysiology involves allostatic load on the hypothalamic-pituitary-adrenal (HPA) axis, a key mediator of the stress response regulating the secretion of glucocorticoids by the adrenal gland (52, 53). In an allostatic model, constant input throughout the life course of an individual will generate learning and adaptive responses, but it may promote ablation of the HPA axis and the emergence of diseases (14). Supporting this idea, variation of cortisol level is observed in the blood of depressive and patients with T2D compared to healthy control participants (54, 55).

Physiologically, insulin elevates adrenocorticotropin and corticosterone hormone levels, promoting HPA axis activation in rats (56). Also, insulin receptor knockdown at the arcuate nucleus of the hypothalamus led to reduced vasopressin response to restraint stress, suggesting that brain insulin resistance could cause disturbances in the HPA axis (14, 56). The hippocampus is proposed to exert negative feedback regulation on the HPA axis (57). Chronic unpredictable stress modulates glucocorticoid and serotonin receptors in the hippocampus of rats, similar to what was observed in the hippocampus of suicidal victims with medical history of depression (58). Interestingly, diabetic rats show decreased expression of hippocampal glucose-dependent type 1 glucocorticoid receptor (59) and lower HPA axis regulation by insulin with decreased response of corticosteroid receptor expression by the hippocampus (60). Collectively, results suggest that brain insulin signaling dysfunction could impair the HPA axis normal response to stress, possibly facilitating the development of depression.

### Tumor Necrosis Factor α (TNFα)

T2D patients have elevated circulating levels of pro-inflammatory markers, in particular of the cytokine tumor necrosis factor α (TNFα) (61). Clinical studies indicate that blood concentrations of the pro-inflammatory cytokine tumor necrosis factor α (TNFα) correlates with depression and impaired performance on memory tests (62, 63). In a Bavarian cohort with history of depression elevated blood levels of TNFα, two isoforms of the soluble TNFα receptor and diabetes were commonly observed (64). In mice, intracerebroventricular administration of TNFα induced depressive-like behavior in the forced swim and tail suspension tests, effects that were counteracted by the ablation of the TNFα receptor 1 (TNFR1) (65). On the other hand, a recent study demonstrated that TNFR1 was involved with anxiety-like behavior analyzed by the open-field test, but not with more related depression tests like the forced swim test (66).

TNFα was shown to contribute to depressive states by modifying the serotonin system. For instance, this cytokine can activate the enzyme indoleamine 2,3-dioxygenase degrading the precursor molecule tryptophan and indirectly decreasing brain serotonin levels (67, 68). This cytokine also promotes blood-brain barrier (BBB) disruption in patients with T2D and in a mouse model of depression (69, 70), which could ultimately lead to loss of the BBB transport regulation of other inflammatory signals, and exacerbate allostatic load to the HPA axis, leading to its dysregulation (71–74).

TNFα promotes insulin resistance by the phosphorylation of insulin receptor substrate 1 (IRS1) on serine residues, via the activation of cellular stress-response kinases, including IκB kinase β (IKKβ), c-Jun N-terminal kinase (JNK), and protein kinase RNA-activated (PKR) (12, 75–78). Increased levels of IRS1 phosphorylated at serine residues are observed in the hippocampus and in the hypothalamus of AD mouse models. In the AD context, activation of stress-response kinases was shown to regulate brain insulin signaling impairment and memory behavior tests (12, 13, 75, 78). TNFα further activates the nuclear factor κ B (NFκB), a transcriptional factor that regulates neuronal survival and the transcription rate of other cytokines, that will convey on insulin signaling impairment (79, 80). The NAc of HFD mice have reduced insulin signaling and increased expression of TNFα. Interestingly, both measures plus depressive-like behavior were counteracted by the administration of probiotics (81).

**Figure 1:**
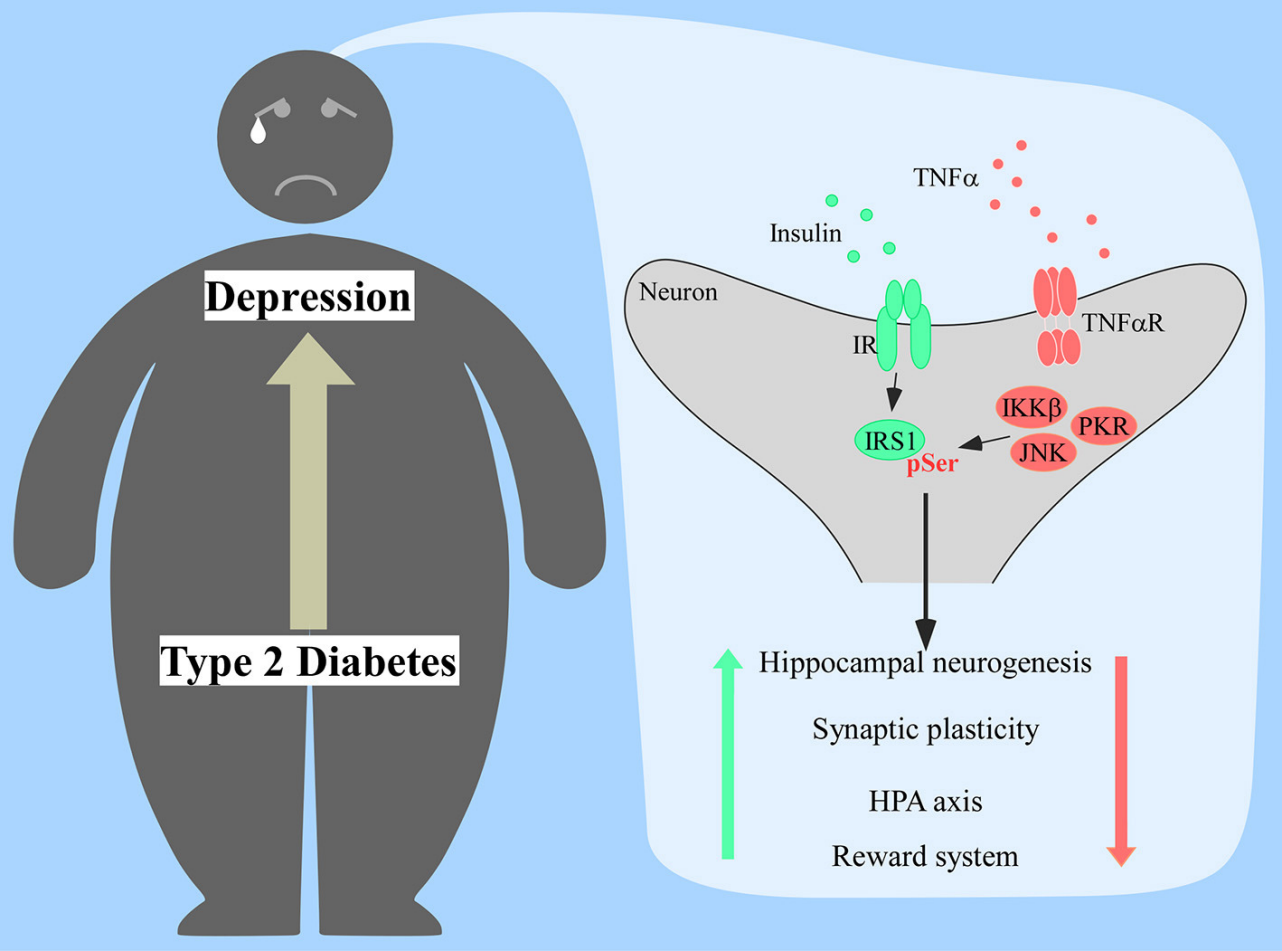
Proposed mechanism of insulin resistance in the brain of diabetic patients prompting the onset of depression. Increased production of tumor necrosis factor α (TNFα) would activate several stress kinases in the brain, including IκB kinase β (IKKβ), c-Jun N-terminal kinase (JNK), and protein kinase RNA-activated (PKR). Activation of those stress pathways leads to the phosphorylation of insulin receptor substrate 1 (IRS1) at serine residues, impacting proper insulin signaling response. Lack of proper central insulin signaling would affect hippocampal neurogenesis, synaptic plasticity, hypothalamic-pituitary-adrenal (HPA) axis response, and the reward system.

Depressive-like behavior was also reported in these models and it was dependent of TNFα signaling and activation of microglial Toll-like receptor 4 (TLR4) (82). Interestingly, TLR4 expression correlates with depression in humans (83, 84) and followed anxiety and depressive-like behavior in mice fed a high-cholesterol diet (85). Since this receptor can be activated by saturated fatty acids (86), which are associated with higher risk of developing T2D (87), a pathway involving TLR4, TNFα, and insulin resistance could be a mechanistic link between T2D and depression yet to be explored (Figure 1).

## Antidepressant Aspects of Antidiabetic Drugs

Due to the heterogeneity of depression and the lack of specific biomarkers, the management of this condition in the primary care setting remains challenging (88, 89) and treatment frequently involves trial and error experimentation. Different types of antidepressants are applied in clinical practices, usually directed to neurotransmitters reuptake and monoamine oxidase inhibition. However, response rates to treatments remain low, with more than 30% of patients with depressive disorders failing to respond to four different antidepressant therapies (90). It is not uncommon to observe cases of treatment-resistant depression despite adequate dosing and duration of antidepressants (91). More effective treatments are required, and some overlapping mechanisms between T2D and depression, suggested above, opens up new avenues for the identification of novel pharmacological targets for the treatment of those comorbid disorders. Regarding this topic, anti-depressant effects of anti-diabetic and anti-inflammatory medications are currently being explored in the field.

Insulin based medications can be considered first-line treatments in T2D in cases of severe basal hyperglycemia or elevated serum glycated hemoglobin. Clinical studies administering intranasal insulin in healthy human subjects have reported improvements in mood and memory (92), as well as better HPA axis response to a social stress test, assessed by decreased saliva and plasma cortisol levels (93). But when evaluated in a cohort of depressive patients, intranasal insulin had no improvements on the depressive scores and neurocognition indexes applied compared to the placebo group (94). Lack of effective response to insulin amongst patients could be related to an intracellular insulin resistance (Figure 1), suggesting that approaches that bypass the first steps of the insulin signaling might show better results.

Liraglutide, another anti-diabetic medication, is an incretin analog that binds to the Glucagon-like peptide 1 receptor and ameliorates insulin signaling. This injectable anti-diabetic medication is capable of crossing the blood-brain barrier (95) and shows positive effects on brain insulin signaling and memory performance on animal models of AD (78, 96, 97). When tested in animal models of depression, liraglutide also had beneficial effects (98, 99). Clinical trials using this drug as a treatment for neurodegenerative diseases, like Alzheimer's disease are ongoing (ClinicalTrials.gov Identifiers: NCT01843075; NCT01469351; NCT02140983). A 4-week pilot study adding liraglutide as a treatment for patients with mood disorders observed better scores on measures of cognitive function compared to baseline (100). But more robust trials involving liraglutide and a placebo group in depressive patients are still warranted.

Metformin is a commonly used treatment for T2D with mechanisms of action not fully understood, but it involves key regulators of cellular energy status, including mitochondrial proteins and the AMP kinase (AMPK) (101, 102). In a cohort of patients with comorbid depression and T2D, metformin was reported to ameliorate depressive behavior when compared to baseline (103). Nonetheless, in a study involving overweight participants with impaired glucose tolerance, metformin had no effect on the Beck Depression Inventory score when compared to the placebo group (104). The effects of metformin in the antidepressant response to sertraline in a group of obese people is currently being evaluated in a phase 4 clinical trial (ClinicalTrials.gov Identifier: NCT00834652).

PPARγ receptor agonists such as thiazolidinediones enhances insulin sensitivity and are used as anti-diabetic drugs (105). Rosiglitazone administration provides antidepressant-like effects in mouse models of depression and T2D (31, 106). Pioglitazone has been evaluated in several clinical trials involving depressed patients and analysis indicated that the drug was more effective in patients with insulin resistance (107–110). Interestingly, pioglitazone effectiveness was age-dependent, being more efficacious in younger subjects (107).

Recent studies have also shown that immunosuppressive agents can improve outcomes in depression. Etanercept and infliximab neutralize TNFα and are currently being used in the treatment of auto-immune disorders (111). In regard to brain diseases, pilot studies administering TNFα inhibitors via intrathecal and perispinal routes had beneficial effects on cognitive measures in AD patients (112–114). But, when delivery subcuteously, etanercept had no beneficial effects on cognition (115), possibly due to restricted transport across the blood-brain barrier (116). A randomized double-blind placebo-controlled trial using an indwelling catheter to deliver infliximab in depressed patients showed no significant changes in Hamilton-Depression (HAM-D) score when compared with the placebo group. However, in patients with a higher inflammatory state, indicated by serum C-Reactive protein (CRP) concentration higher than 5 mg/L, infliximab improved HAM-D scores compared to placebo (117). Since the treatment decreased circulating CRP levels within the responder vs. the non-responder group, and the limitation of infliximab to reach the brain, the beneficial effect of the drug might have been driven by an overall decrease of peripheral inflammatory markers.

Finally, changes in the gut microbiota are associated with stress disorders (118). Probiotics can modulate the HPA axis (119), neurotransmitters (120), inflammatory markers and, as previously mentioned, insulin signaling in the brain (81). Probiotics are emerging as promising treatments for depression, showing positive results in different clinical studies [for a systematic review see (121)].

## Conclusion

The association between depression and diabetes is supported by several evidences, but the mechanistic links between both diseases are still emerging. The development of brain insulin resistance is a possible candidate connecting both diseases, but further studies focusing on this issue are warranted in the field. Unraveling this connection is a matter of a great value in order to pursue alternative treatments or to optimize anti-depressants response.

## Author Contributions

NL: development of the subject matter, drafting of the article, conception, and design of the figure, critical revision of the article, final approval of the version to be published; ML: development of the subject matter, drafting of the article, critical revision of the article, final approval of the version to be published; CS and DM: critical revision of the article, final approval of the version to be published; RM and FD: development of the subject matter, drafting of the article, critical revision of the article, final approval of the version to be published.

### Conflict of Interest Statement

The authors declare that the research was conducted in the absence of any commercial or financial relationships that could be construed as a potential conflict of interest.
